# Particulate Matter Exposure Impairs Systemic Microvascular Endothelium-Dependent Dilation

**DOI:** 10.1289/ehp.7001

**Published:** 2004-06-23

**Authors:** Timothy R. Nurkiewicz, Dale W. Porter, Mark Barger, Vincent Castranova, Matthew A. Boegehold

**Affiliations:** ^1^Department of Physiology and Pharmacology and; ^2^Center for Interdisciplinary Research in Cardiovascular Sciences, West Virginia University School of Medicine, Morgantown, West Virginia, USA; ^3^Pathology and Physiology Research Branch, Health Effects Laboratory Division, National Institute for Occupational Safety and Health, Morgantown, West Virginia, USA

**Keywords:** arteriole, endothelium, nitric oxide, particulate matter, residual oil fly ash, ROFA, spinotrapezius muscle, systemic microcirculation, titanium dioxide

## Abstract

Acute exposure to airborne pollutants, such as solid particulate matter (PM), increases the risk of cardiovascular dysfunction, but the mechanisms by which PM evokes systemic effects remain to be identified. The purpose of this study was to determine if pulmonary exposure to a PM surrogate, such as residual oil fly ash (ROFA), affects endothelium-dependent dilation in the systemic microcirculation. Rats were intratracheally instilled with ROFA at 0.1, 0.25, 1 or 2 mg/rat 24 hr before experimental measurements. Rats intratracheally instilled with saline or titanium dioxide (0.25 mg/rat) served as vehicle or particle control groups, respectively. *In vivo* microscopy of the spinotrapezius muscle was used to study systemic arteriolar dilator responses to the Ca^2+^ ionophore A23187, administered by ejection via pressurized micropipette into the arteriolar lumen. We used analysis of bronchoalveolar lavage (BAL) samples to monitor identified pulmonary inflammation and damage. To determine if ROFA exposure affected arteriolar nitric oxide sensitivity, sodium nitroprusside was iontophoretically applied to arterioles of rats exposed to ROFA. In saline-treated rats, A23187 dilated arterioles up to 72 ± 7% of maximum. In ROFA- and TiO_2_-exposed rats, A23187-induced dilation was significantly attenuated. BAL fluid analysis revealed measurable pulmonary inflammation and damage after exposure to 1 and 2 mg ROFA (but not TiO_2_ or < 1 mg ROFA), as evidenced by significantly higher polymorphonuclear leukocyte cell counts, enhanced BAL albumin levels, and increased lactate dehydrogenase activity in BAL fluid. The sensitivity of arteriolar smooth muscle to NO was similar in saline-treated and ROFA-exposed rats, suggesting that pulmonary exposure to ROFA affected endothelial rather than smooth muscle function. A significant increase in venular leukocyte adhesion and rolling was observed in ROFA-exposed rats, suggesting local inflammation at the systemic microvascular level. These results indicate that pulmonary PM exposure impairs systemic endothelium-dependent arteriolar dilation. Moreover, because rats exposed to < 1 mg ROFA or TiO_2_ did not exhibit BAL signs of pulmonary damage or inflammation, it appears that PM exposure can impair systemic microvascular function independently of detectable pulmonary inflammation.

Evidence from industrialized countries indicates that acute exposure to airborne pollutants, such as solid particulate matter (PM), is associated with an increased risk of morbidity and mortality (Dockery 2001; Fairley 1990; Samet et al. 2000a, 2000b). The collective implication of this evidence is that PM affects tissues and organs outside the respiratory tract, as evidenced by the occurrence of cardiovascular dysfunction on high pollution days (Goldberg et al. 2000, 2001; Samet et al. 2000a). Although the epidemiologic evidence is convincing, the biologic mechanisms by which PM evokes systemic effects remain to be defined.

Only recently have scientists started to recognize that the systemic effects of PM may be as important as the pulmonary effects. Despite its obvious importance in regulating the convective delivery of cells and molecules to all tissues, and in the etiology of most cardiovascular diseases, no research has investigated how systemic microvascular function is affected by pulmonary PM exposure. However, the following results suggest that systemic microvascular effects of pulmonary PM exposure are possible. Constriction of the brachial artery has been observed in humans exposed to PM and ozone (Brook et al. 2002). PM elicits vasorelaxation in aortic rings that had been precontracted with phenylephrine but has no effect on small isolated arteries (Bagate et al. 2004; Knaapen et al. 2001). Although intriguing, conclusions from these latter findings are limited, because isolation of any tissue and subsequent exposure to PM *in vitro* assumes that PM must access the systemic circulation and interact directly with the vasculature.

Endothelial products can have potent effects on microvascular smooth muscle tone. Ulrich et al. (2002) found that pulmonary exposure to PM caused a 60% decrease in endothelin-1 and angiotensin-converting enzyme mRNA levels in lung tissue. They interpreted this result as an indication of possible endothelial damage in pulmonary blood vessels. However, the effect of pulmonary exposure to PM on endothelial cell function in the systemic microcirculation has not been evaluated.

Although several investigations have documented systemic responses to PM exposure, the underlying mechanisms of action remain unclear. However, inflammation appears to be a common denominator in the studies that report a systemic effect of PM exposure (Gardner et al. 2000; Moyer et al. 2002; Salvi et al. 1999; Tan et al. 2000; van Eeden et al. 2001). Cultured human alveolar macrophages (AMs) produce tumor necrosis factor (TNF-α) and proinflammatory cytokines such as granulocyte-macrophage colony-stimulating factor, interleukin-6 (IL-6), and IL-1 after phagocytosing PM. Additionally, circulating IL-1 is elevated in humans exposed to PM (van Eeden et al. 2001). Pulmonary production of such mediators may induce systemic responses. Indeed, TNF-α, IL-6, and IL-1 can stimulate microvascular dilation, alter leukocyte dynamics and increase microvascular permeability (Baudry et al. 1996; Brian and Faraci 1998; Kunkel et al. 1998; Matsuki and Duling 2000; Vicaut et al. 1991). Acute exposure to PM accelerates the transit of polymorphonuclear leukocytes (PMNLs) from bone marrow, whereas chronic exposure increases the size of the bone marrow PMNL pool (van Eeden and Hogg 2002). Blood samples from healthy humans exposed to PM reveal a systemic inflammatory response in the form of elevated immature PMNL (Tan et al. 2000), neutrophils, and platelets (Salvi et al. 1999). In addition to elevating circulating PMNLs, PM exposure has also been shown to accelerate the formation of athero-sclerotic lesions and increase plaque cell turnover (Suwa et al. 2002). Collectively, these systemic inflammatory events associated with PM exposure have been proposed to be linked to cardiovascular dysfunction in compromised individuals. However, specific changes and/or effects of PM exposure at the systemic microvascular level, where the majority of peripheral resistance resides (Renkin 1984; Zweifach et al. 1981; Zweifach and Lipowsky 1984) and vascular dysfunction can be lethal, have not been evaluated.

Microvascular resistance is responsible for the largest dissipation of pressure across the systemic circulation (Renkin 1984; Zweifach et al. 1981; Zweifach and Lipowsky 1984). The resistance of a given microvascular network is a function of network branching patterns, vessel anatomy, and vessel density (Zweifach et al. 1981). Because arteriolar resistance is a highly dynamic state that is chiefly determined by internal vascular diameter, it is acutely dependent on a delicate balance between local, humoral, and neural stimuli, as well as the microvascular wall anatomy. Disruption, impairment, or imbalance of these stimuli, often accompanied by changes in wall structure, have been well characterized in cardiovascular diseases such as hypertension and ischemia, with myocardial infarction and stroke being the most lethal (Baumbach et al. 2002; Hein et al. 2003; Prewitt et al. 2002; Schwartzkopff et al. 1992, 1998; Suzuki et al. 1994).

Based on the prevalence of cardiovascular dysfunction on high pollution days, we hypothesized that impairment of normal microvascular endothelial cell function constitutes an important component of the systemic effects associated with pulmonary PM exposure. The purpose of this study was to determine the effects of pulmonary PM exposure on systemic endothelium-dependent arteriolar dilation. Intratracheal (IT) instillation and bronchoalveolar lavage (BAL) were used in conjunction with intravital microscopy to study the effects of residual oil fly ash (ROFA) exposure on microvascular function in the rat spinotrapezius muscle. The spinotrapezius muscle preparation has been used for > 30 years as an experimental model to evaluate physiologic and pathophysiologic phenomena within the microcirculation (Bailey et al. 2000; Gray 1973). This preparation has been an essential tool in the fundamental understanding of capillary network development (Skalak and Schmid-Schonbein 1986); neurogenic, humoral, and myogenic control of microvascular resistance (Lash and Shoukas 1991; Nakamura and Prewitt 1991; Nurkiewicz and Boegehold 1998); and the physiologic roles of oxygen, nitric oxide, and calcium (Ca^2+^) in microcirculation (Linderman and Boegehold 1999; Pries et al. 1995; Toth et al. 1998). Additionally, the spinotrapezius muscle preparation has been extensively used to characterize pathophysiologic microvascular consequences of chronic diseases such as diabetes (Kindig et al. 1998), hypertension (Boegehold 1991; Nurkiewicz and Boegehold 1998; Zweifach et al. 1981), and heart failure (Kindig et al. 1999). Arterioles of the first branching order were studied because these vessels, together with their upstream feed arteries, are responsible for approximately 60% of total spinotrapezius muscle vascular resistance and therefore are of major importance for local blood flow regulation (Boegehold 1991).

## Experimental Objectives

### Objective 1.

Our first objective was to determine if pulmonary exposure to PM has an effect on systemic microvascular function. Rats were exposed by IT instillation to various doses of ROFA or saline. At 24 hr postexposure, we evaluated systemic microvascular function by intravital microscopy of arterioles in the spinotrapezius muscle. Microvascular function was determined by measurement of resting arteriolar diameter and tone and by responsiveness to dilation dependent on the calcium ionophore A23187.

### Objective 2.

Our second objective was to determine if the effects of pulmonary exposure to PM on systemic microvascular function were due to modification of endothelial or smooth muscle function. Rats were exposed to PM by IT instillation, and arteriolar smooth muscle sensitivity to NO was determined by iontophoretically applying the NO donor sodium nitroprusside (SNP) to the exterior arteriolar wall and measuring dilation.

### Objective 3.

Our third objective was to determine if the effects of pulmonary exposure to PM on systemic microvascular function were dependent on pulmonary and/or microvascular inflammation. Rats were exposed to PM by IT instillation. Pulmonary inflammation and damage were measured 24 hr postexposure as BAL cell counts and activity, acellular BAL albumin levels, and BAL fluid lactate dehydrogenase (LDH) activity. Microvascular inflammation was monitored microscopically 24 hr postexposure by measuring systemic leukocyte adhesion and rolling in first-order venules of the spinotrapezius muscle.

## Materials and Methods

### ROFA preparation.

ROFA was collected from a precipitator at Boston Edison Company (Mystic Power Plant #4, Everett, MA). ROFA particle size and elemental composition from this source have been previously characterized (Antonini et al. 2002; Roberts et al. 2004). ROFA particles were of respirable size with a count mean diameter of 2.2 μm (as determined by scanning electron microscopy (JSM 5600 SEM; JEOL Ltd., Peabody, MA). The major metal contaminants were iron, aluminum, vanadium, nickel, calcium, and zinc. The main soluble metals were aluminum, nickel, and calcium. Vanadium comprised < 1% of the soluble metals in ROFA samples from this source. ROFA (suspended in sterile saline) was sonicated for 1 min with a Sonifer 450 cell disruptor (Branson Ultrasonics, Danbury, CT) before IT instillation.

### IT instillation.

Male Sprague-Dawley rats (7–8 weeks of age) were lightly anesthetized by an intraperitoneal (ip) injection of sodium methohexitol (Brevital) and instilled with ROFA (0.1, 0.25, 1, or 2 mg/rat, IT, in a 300 μL sterile saline suspension) according to the method of Brain et al. (1976). Rats in the vehicle control group were IT dosed with 300 μL sterile saline. Rats in the particle control group were dosed with titanium dioxide (0.25 mg/rat, IT, in a 300 μL sterile saline suspension). The higher ROFA doses chosen for these experiments (1 mg and 2 mg) have been previously shown to produce significant pulmonary inflammation (Antonini et al. 2002) and fall within the range of concentrations consistently used in other animal studies evaluating pulmonary responses to ROFA (Antonini et al. 2002; Dreher et al. 1997; Gavett et al. 1997; Kodavanti et al. 1998). After IT instillation, all rats were allowed to recover for 24 hr before subsequent BAL or intravital microscopy experiments.

### Collection of BAL samples and blood for measurement of systemic and pulmonary inflammation.

Rats were euthanized with sodium pentobarbital (≥100 mg/kg, ip). A tracheal cannula was inserted, and BAL was performed through the cannula using ice-cold Ca^2+^/Mg^2+^-free phosphate-buffered saline (PBS). The first lavage was 6 mL and was kept separate from the rest of the lavage sample. Subsequent lavages used 8 mL PBS until a total of 80 mL lavage fluid was collected. The samples were centrifuged (650 × *g*, 5 min at 4°C), and the acellular first lavage supernatant (BAL fluid) was aspirated and saved for LDH activity and albumin protein assays. Cells were combined, resuspended in HEPES-buffered medium (10 mM *N*-[2-hydroxyethyl]piperazine-*N*′-[2-ethanesulfonic acid], 145 mM NaCl, 5 mM KCl, 1 mM CaCl_2_, and 5.5 mM d-glucose, pH 7.4) and centrifuged a second time (650 × *g*, 5 min, 4°C). The cells were then resuspended in HEPES-buffered medium, and cell counts were determined with an electronic cell counter equipped with a cell-sizing attachment (Porter et al. 2002). Whole blood was drawn from a carotid cannula (inserted for intravital microscopy experiments, described below) using a Vacutainer blood collection tube with sodium ethylenediamine tetraacetate as an anticoagulant. Blood cell differentials were determined with a Cell-Dyne 3500R hematology cell counter (Abbott Diagnostics, Abbott Park, IL) (Porter et al. 2001).

### BAL fluid LDH activity and albumin protein assays.

All measurements used for the current study were made on the day that samples were taken, and any remaining samples were frozen for long-term storage (−80°C). BAL fluid LDH activities were determined as a marker of cytotoxicity and were measured by monitoring the LDH catalyzed oxidation of pyruvate coupled with the reduction of nicotinamide adenine dinucleotide at 340 nm using a commercial assay kit (LDH Reagent; Roche Diagnostic Systems, Montclair, NJ) and a Cobas Fara II Analyzer (Roche Diagnostic Systems), as previously described (Porter et al. 2002). BAL fluid albumin concentrations were determined as an indicator of the integrity of the blood–pulmonary epithelial cell barrier. BAL fluid albumin was measured colorimetrically at 628 nm based on albumin binding to bromcresol green using a commercial assay kit (Albumin BCG diagnostic kit; Sigma Chemical Co., St. Louis, MO) and a Cobas Fara II Analyzer, as previously described (Porter et al. 2002).

### AM chemiluminescence.

AM chemiluminescence was determined as an indicator of reactive oxygen species production by AM. The use of unopsonized zymosan in the chemiluminescence assay allows only AM chemiluminescence to be measured, because unopsonized zymosan stimulates AM chemiluminescence but not PMNL chemiluminescence. The assay was conducted in a total volume of 0.25 mL HEPES-buffered media. Resting AM chemiluminescence was determined by incubating 1.0 × 10^6^ AM/mL at 37°C for 20 min, followed by the addition of 5-amino-2,3-dihydro-1,4-phthalazinedione (luminol) to a final concentration of 0.08 μg/mL followed by the measurement of chemiluminescence. To determine zymosan-stimulated chemiluminescence, unopsonized zymosan (Sigma) was added to a final concentration of 2 mg/mL immediately before the measurement of chemiluminescence. All chemiluminescence measurements were made with an automated luminometer (Autolumat LB 953; Berthold, Gaithersburg, MD) at 390–620 nm for 15 min. The integral of counts per minute versus time was calculated. Zymosan-stimulated chemiluminescence was calculated as the counts per minute in the zymosan-stimulated sample minus the counts per minute in the resting sample. NO-dependent chemiluminescence was determined by subtracting the zymosan-stimulated chemiluminescence from cells pre-incubated with 1 mM 1400W, an inhibitor of NO synthase, from the zymosan-stimulated chemiluminescence from AM without 1400W.

### Intravital microscopy.

Male Sprague-Dawley rats (7–8 weeks of age) were anesthetized with sodium thiopental (100 mg/kg ip) and placed on a heating pad to maintain a 37°C rectal temperature. The trachea was intubated to ensure a patent airway, and the right carotid artery was cannulated to measure arterial pressure. The right spinotrapezius muscle was then exteriorized for microscopic observation, leaving its innervation and all feed vessels intact. After exteriorization, the muscle was gently secured over an optical pedestal at its *in situ* length. The muscle was next enclosed in a tissue bath for transillumination and observation. In Sprague-Dawley rats of this age, the spinotrapezius muscle weighs 285 ± 56 mg (mean ± SE) and has a surface area of 192 ± 28 mm^2^ (Linderman and Boegehold 1996). Throughout the surgery and subsequent experimental period, the muscle was continuously superfused with an electrolyte solution (119 mM NaCl, 25 mM NaHCO_3_, 6 mM KCl, and 3.6 mM CaCl_2_), warmed to 35°C, and equilibrated with 95% N, 2–5% CO_2_ (pH 7.35–7.40). Superfusate flow rate was maintained at 4–6 mL/min to minimize equilibration with atmospheric oxygen (Boegehold and Bohlen 1988).

The animal preparation was then transferred to the stage of an intravital microscope coupled to a CCD (charge-coupled device) video camera. Observations were made with a 20× water immersion objective (final video image magnification, 1,460×). One to three microvessels were studied per rat. Video images were displayed on a high-resolution color video monitor and videotaped for off-line analysis. During videotape replay, arteriolar inner diameters were measured with a video caliper (Cardiovascular Research Institute, Texas A&M University, College Station, TX), and venular leukocyte adhesion was monitored.

### Experimental protocol 1.

In the first series of experiments, arteriolar endothelium-dependent dilation was evaluated in the four ROFA groups and the saline and TiO_2_ groups by assessing the capacity for Ca^2+^-dependent endothelial NO formation in response to intraluminal infusion of the calcium ionophore A23187 (Sigma). Glass micropipettes were beveled to a 23–25° angle and a 2–4 μm inner tip diameter. One mg of A23187 was dissolved in 50 μL dimethyl sulfoxide and then serially diluted in PBS to produce a 10^−7^ M solution that was loaded into the micropipettes. A23187 increases endothelial NO synthase activity, which produces NO and therefore relaxes exposed vessels (Schneider et al. 2003). The micropipette was inserted into the lumen of the selected arteriole approximately 100 μm upstream from the diameter measurement site, and A23187 was then infused directly into the flow stream. A Picospritzer II pressure system (General Valve Corporation, Fairfield, NJ) was used to continuously infuse A23187 for 2-min periods at net ejection pressures of 5, 10, 20, and 40 psi. In preliminary tests, we verified that the amount of agonist ejected from the pipette tip is directly proportional to the ejection pressure. The order in which the different ejection pressures were applied was randomized in each experiment, and a 2-min recovery period followed each ejection. Given the resistance of the pipette tips, the fluid volumes ejected at these pressures are relatively small and increase total arteriolar volume flow by no more than 10% (Nurkiewicz TR, Boegehold MA, unpublished observations). At the end of each experiment, adenosine (ADO) was added via a syringe pump to the superfusate at a final concentration of 10^−4^ M to fully dilate the microvascular network and determine the passive diameter of each arteriole studied. We have previously shown that the magnitude of arteriolar dilation induced by 10^−4^ M ADO is not further augmented by subsequent exposure to SNP plus nifedipine in a Ca^2+^-free superfusate, indicating that 10^−4^ M ADO completely abolishes arteriolar tone in the exteriorized spinotrapezius muscle without altering systemic arterial pressure (Nurkiewicz and Boegehold 2000).

In a separate series of experiments, the contribution of NO to endothelium-dependent arteriolar dilation in the spinotrapezius muscle was evaluated by inhibiting local NO synthesis with N^G^-monomethyl-l-arginine (L-NMMA). Rats in this group of experiments were of the same age and weight range as those used in other protocols but did not receive IT saline. A23187 was infused into arterioles first under the normal superfusate at the four ejection pressures and again in the presence of L-NMMA in the superfusate. A syringe pump was used to continuously infuse L-NMMA at 0.4 mL/min into the superfusate delivery line. The stock L-NMMA concentration was adjusted to produce a final superfusate concentration of 10^−4^ M. We have previously shown that, in this vascular bed after 15 min of preincubation, L-NMMA at this concentration maximally inhibits the dilation of spinotrapezius muscle arterioles to acetylcholine (Boegehold 1995) and that L-NMMA is a specific inhibitor of NO synthesis (Nurkiewicz and Boegehold 1999).

### Experimental protocol 2.

To evaluate arteriolar responsiveness to NO, the NO donor SNP was iontophoretically applied to individual arterioles in rats exposed to either saline or 0.25 mg ROFA. Glass micropipettes were prepared as above and filled with a 0.05 M solution of SNP in distilled water. The pipette tip was placed in light contact with the arteriolar wall, and a current programmer (model 260; World Precision Instruments, New Haven, CT) was used to deliver continuous 2-min ejection currents of 5, 10 and 20 nA. A recovery period of at least 2 min followed each application. The order of the 5- and 10-nA ejection currents was randomized, but the 20-nA ejection current was always performed last because of a considerably slower recovery from this stimulus. At the end of each experiment, ADO was added to the superfusate at a final concentration of 10^−4^ M to determine the passive diameter of each arteriole studied.

### Experimental protocol 3.

Adhering or rolling leukocytes in first-order venules of rats exposed to either saline or 2 mg ROFA were quantified to characterize potential microvascular inflammation. Leukocytes that were either stationary or moving but in constant contact with the venular wall for at least 200 μm were counted for 1 min in each venule studied.

### Data and statistical analysis.

Arteriolar diameter (*D*, in micrometers) was sampled at 10-sec intervals during all control and infusion periods. Resting vascular tone was calculated for each vessel as follows: tone = [(*D*_pass_ − *D_c_*)/*D*_pass_] × 100, where *D*_pass_ is passive diameter under ADO and *D_c_* is the diameter measured during the control period. A tone of 100% represents complete vessel closure, whereas 0% represents the passive state. For comparisons of arteriolar responses to A23187 infusion among different treatments and experimental groups, responses were normalized as follows: percent change from control = [(*D*_ss_/*D_c_*) − 1] × 100, where *D*_ss_ is the steady-state diameter during A23187 exposure. All data are reported as mean ± SE. Statistical analysis was performed by commercially available software (Sigmastat; SPSS, Chicago, IL). One-way repeated-measures analysis of variance (ANOVA) was used to determine the effect of a treatment within a group or differences among groups. Two-way repeated measures ANOVA was used to determine the effects of group, treatment, and group–treatment interactions on measured variables. For all ANOVA procedures, the Student-Newman-Keuls method for post hoc analysis was used to isolate pair wise differences among specific groups. Significance was assessed at the 95% confidence level (*p* < 0.05) for all tests.

## Results

The general characteristics of rats used for intravital microscopy experiments are reported in Table 1. At the time of study, mean age, body weight, and arterial pressure were not significantly different in the experimental groups. Rats used for BAL data were of the same age and body weight as those reported in Table 1 (data not shown). Resting variables of all arterioles studied are reported in Table 2. Resting arteriolar diameters were not significantly different among the experimental groups. Passive diameters (in the presence of ADO) in the 1-mg ROFA group were significantly greater than those in the 0.25-mg TiO_2_ and 0.25-mg ROFA groups; however, none of the particle groups was different from saline. Resting arteriolar tone was not different among the experimental groups.

The effects of pulmonary exposure to ROFA on BAL fluid and pulmonary cells are reported in Table 3. AMs were not significantly different in the experimental groups. However, ROFA exposure did cause dose-dependent changes in other indicators of pulmonary inflammation and damage. PMNL counts were significantly elevated in the 1- and 2-mg ROFA groups versus those in the saline and 0.25-mg TiO_2_ groups. Additionally, PMNL counts in the 1- and 2-mg ROFA groups were significantly greater than those in the 0.25- and 0.1-mg ROFA groups, which did not differ from either the saline or particle control (TiO_2_) groups. BAL fluid albumin was significantly elevated in the 1- and 2-mg ROFA groups versus that in the saline and 0.25-mg TiO_2_ groups. BAL fluid albumin in the 1- and 2-mg ROFA groups was also significantly greater than that in the 0.25- and 0.1-mg ROFA groups, which did not differ from either the saline or particle control (TiO_2_) groups. BAL fluid LDH in the 1- and 2-mg ROFA groups was significantly higher than that in the saline and 0.25-mg ROFA groups. BAL fluid LDH in the 1-mg ROFA group also was significantly greater than that in the 0.25-mg TiO_2_ group. In three rats (one exposed to 0.1 mg ROFA and two exposed to 0.25 mg TiO_2_), insertion of the tracheal cannula during BAL caused bleeding that distorted the albumin and LDH data in these rats. Therefore, BAL fluid data from those animals were omitted from Table 3. Total zymosan-stimulated AM chemiluminescence was significantly greater in the 2-mg ROFA group than that in the saline, TiO_2_, 1-mg, or 0.1-mg ROFA groups. NO-dependent AM chemiluminescence was significantly higher in the 2-mg ROFA group than that in all other groups.

**Figure 1:**
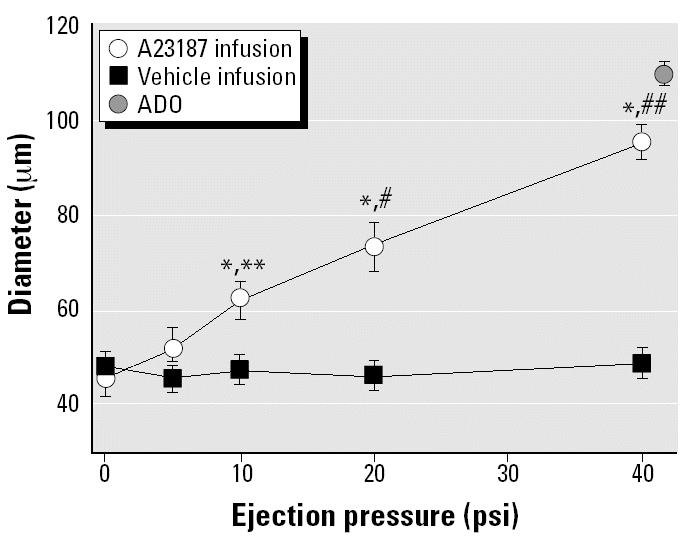
Intraluminal A23187 infusion produces graded, near-maximal arteriolar dilation in rats exposed to IT saline; the vehicle is not inherently vasoactive. Values are mean ± SE. For A23187 infusion, *n* (number of arterioles studied) = 10; for vehicle infusion, *n* = 7.
*p < 0.05 vs. vehicle at the same ejection pressure. **p < 0.05 vs. 5 psi response. ^#^*p* < 0.05 vs. 10 psi response. ^##^*p* < 0.05 vs. 20 psi response.

In spinotrapezius muscle arterioles, A23187 infusion produced dose-dependent dilation that was near maximal at the highest ejection pressure in the IT-saline control group (Figure 1). This dilation was not an artifact of ejection pressure because infusion of the vehicle did not elicit a significant vasoactive response at any of the ejection pressures used in this study.

**Figure 2:**
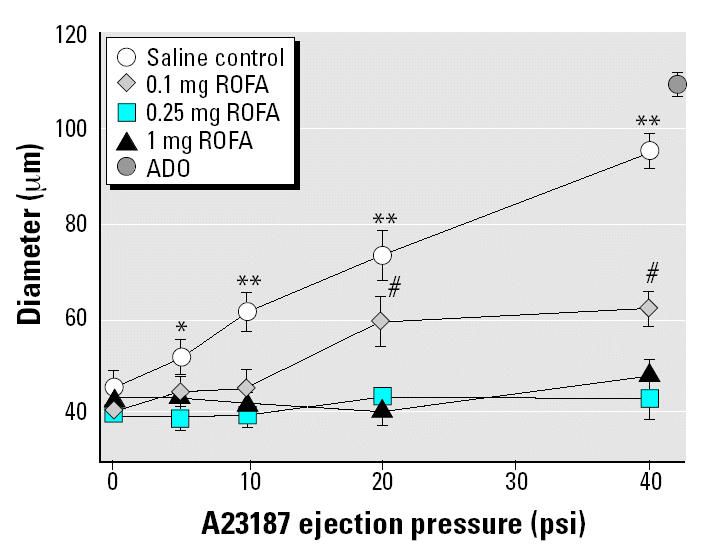
ROFA exposure impairs or abolishes spinotrapezius muscle arteriolar responsiveness to intraluminal A23187. Values are mean ± SE. For saline control, *n* (number of arterioles studied) = 10; for 0.1 mg ROFA, *n* = 9; for 0.25 mg ROFA, *n* = 8; for 1 mg ROFA, *n* = 8.
*p < 0.05 vs. 0.25 mg ROFA. **p < 0.05 vs. 0.1, 0.25, and 1 mg ROFA. ^#^*p* < 0.05 vs. 0.25 mg and 1 mg ROFA.

IT treatment of rats with 0.25 mg or 1 mg ROFA completely inhibited A23187-induced dilation of spinotrapezius muscle arterioles (Figure 2). Arterioles in rats exposed to 2 mg ROFA also failed to respond to A23187 infusion (data omitted from Figure 2 for clarity). In rats exposed to 0.1 mg ROFA, A23187 infusion produced an intermediate dilation at 20 and 40 psi that was significantly greater than that observed in rats exposed to 0.25 mg, 1 mg, or 2 mg ROFA but was less than that in IT-saline control rats. Therefore, pulmonary exposure to ROFA inhibited systemic microvascular function in a dose-dependent manner.

**Figure 3:**
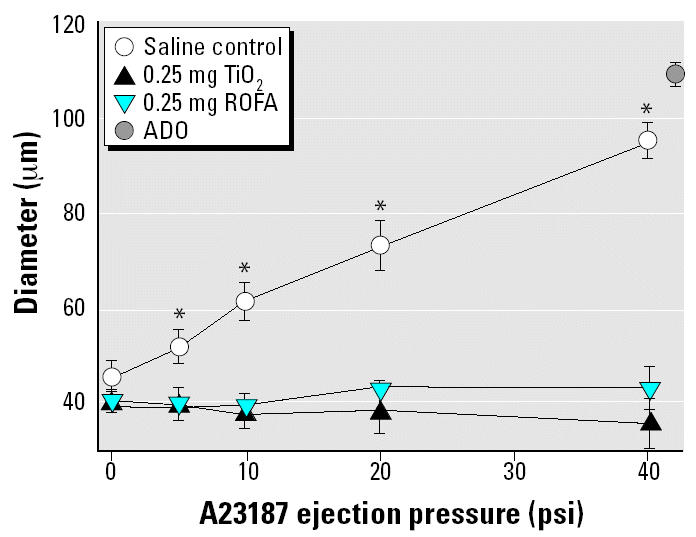
TiO_2_ exposure abolishes spinotrapezius muscle arteriolar responsiveness to intraluminal A23187. Values are mean ± SE. For saline control, *n* (number of arterioles studied) = 10; for 0.25 mg TiO_2_, *n* = 8; for 0.25 mg ROFA, *n* = 8.
*p < 0.05 vs. 0.25 mg TiO_2_ and 0.25 mg ROFA.

To determine if the inhibition of systemic vasodilation after pulmonary exposure to ROFA was substance specific, arteriolar responses to A23187 after IT instillation of a noninflammatory particle (TiO_2_) were evaluated. A23187 infusion had no effect on arteriolar diameter in rats exposed to 0.25 mg TiO_2_ (Figure 3). The arteriolar responses to A23187 infusion between rats exposed to 0.25 mg ROFA and 0.25 mg TiO_2_ were not different. Therefore, inhibition of systemic microvascular function in response to pulmonary PM exposure does not appear to depend on the pulmonary toxicity of the particle.

**Figure 4:**
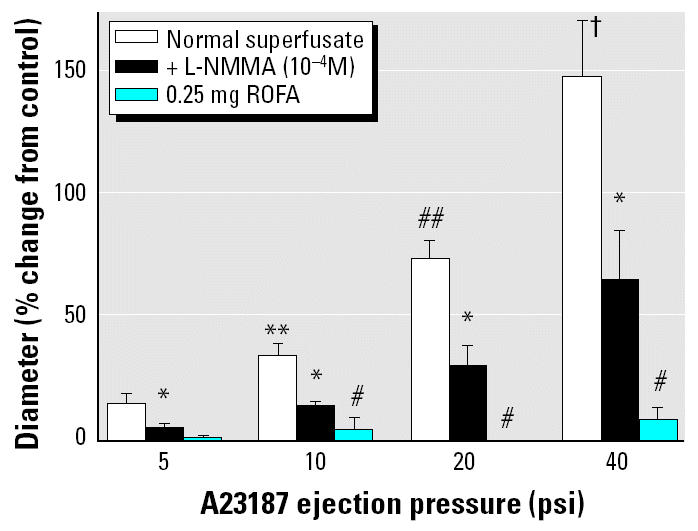
ROFA exposure impairs both NO-dependent and NO-independent arteriolar responsiveness to intraluminal A23187 in the spinotrapezius muscle. NO synthase was competitively inhibited by simultaneous superfusion with L-NMMA (10^−4^ M final superfusate concentration). Values are mean ± SE. For normal superfusate and with L-NMMA, *n* (number of arterioles studied) = 8; for 0.25 mg ROFA, *n* = 8.
*p < 0.05 vs. normal superfusate. **p < 0.05 vs. 5 psi response. ^#^*p* < 0.05 vs. +L-NMMA. ^##^*p* < 0.05 vs. 10 psi response. ^†^*p* < 0.05 vs. 20 psi response.

In Figure 4, the effect of A23187 infusion on arteriolar diameter is expressed in terms of percent change from control. A comparison is made between arteriolar responses under the normal superfusate, during L-NMMA superfusion, and in rats exposed to 0.25 mg ROFA. The normal superfusate and L-NMMA data shown here were previously collected from spinotrapezius muscle first-order arterioles in Sprague-Dawley rats of the same age and weight range as those used in the present study but did not receive IT saline (Nurkiewicz and Boegehold 2004). During L-NMMA superfusion, arteriolar dilation in response to A23187 was significantly decreased at each ejection pressure, indicating that > 50% of the arteriolar dilation due to A23187 infusion is NO dependent. The residual response to A23187 that persists in the presence of L-NMMA is NO independent. In rats exposed to 0.25 mg ROFA, arteriolar responses to A23187 at 10, 20, and 40 psi were significantly less than those responses observed during superfusion with L-NMMA. Thus, IT exposure to ROFA inhibits both NO-dependent and NO-independent components of A23187-induced dilation of systemic arterioles.

**Figure 5:**
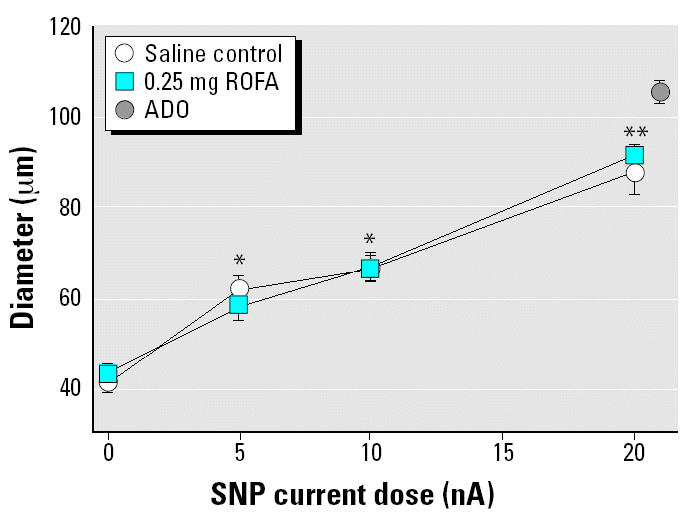
ROFA exposure does not alter spinotrapezius muscle arteriolar responsiveness to SNP. Values are mean ± SE. For saline control, *n* (number of arterioles studied) = 8; for 0.25 mg ROFA, *n* = 9.
*p < 0.05 vs. 0 nA in both groups. **p < 0.05 vs. 10 nA in both groups.

Figure 5 displays arteriolar responsiveness to iontophoretically applied SNP in rats exposed to either saline or 0.25 mg ROFA. The arteriolar responses to SNP at each current dose represent significant dilations from control diameter and were dose dependent. The arteriolar responses to SNP in the IT-saline control group and ROFA group were not different at any current dose. Thus, pulmonary exposure to ROFA did not alter the responsiveness of arteriolar smooth muscle to the dilator effects of NO.

**Figure 6:**
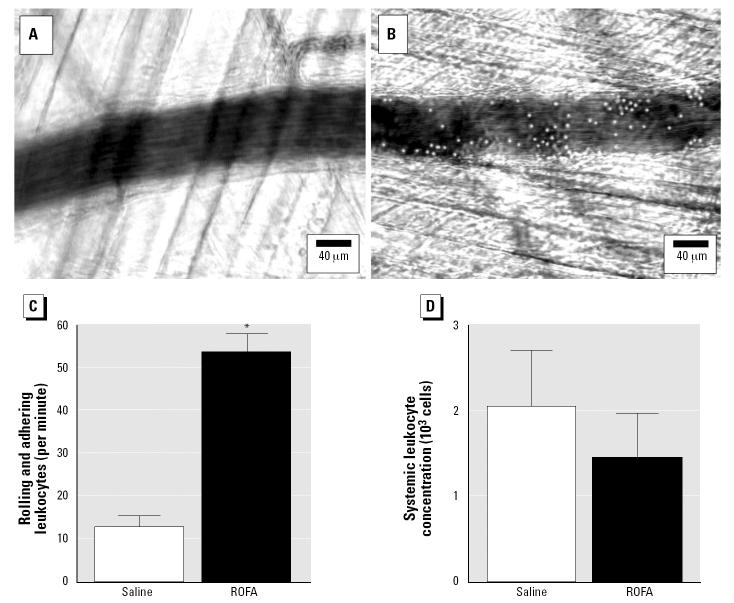
ROFA exposure increases venular leukocyte rolling and adhesion in the spinotrapezius muscle. Abbreviations: *n*, number of venules studied; *N*, number of rats studied. (*A*) Representative venule in an IT-saline control rat. (*B*) Representative venule in a rat exposed to 2 mg ROFA. (*C*) Rolling and adhering venular leukocytes observed per minute in a 200-μm segment [for IT-saline control, *n* = 15; for 2 mg ROFA, *n* = 15]. (*D*) Systemic leukocyte concentration in saline control rats (*N* = 3) and rats exposed to 2 mg ROFA (*N* = 3). Values in (*C*) and (*D*) are mean ± SE.
*p < 0.05 vs. IT-saline control.

Microvascular leukocyte activity in the spinotrapezius muscle is characterized in Figure 6. Such characterization involved monitoring the number of adhering and rolling leukocytes in venules that were closely paired to the arterioles of interest. Figure 6A shows a representative venule in a saline-treated rat. Figure 6B shows a representative venule in a rat exposed to 2 mg ROFA, where an increased leukocyte presence is clearly visible. Figure 6C indicates that leukocyte adherence and rolling was 4-fold greater in venules of ROFA-exposed rats than in saline-treated rats. This result is consistent with a local inflammatory response at the systemic microvascular level. Figure 6D indicates that systemic leukocyte concentrations were not different between ROFA-exposed and saline-treated rats.

## Discussion

To our knowledge, this is the first study to demonstrate a significant effect of acute pulmonary PM exposure at the systemic microvascular level. There are four prominent observations within this study.

First, pulmonary PM exposure significantly impairs A23187-induced arteriolar endothelium-dependent dilation in the rat spinotrapezius muscle (Figures 2 and 3). In saline-treated rats, this microvascular response to intraluminal infusion of A23187 was dose dependent and near the maximal level of dilation. Resting arteriolar diameter was unchanged after ROFA or TiO_2_ exposure (Table 2). However, A23187-induced arteriolar endothelium-dependent dilation was either significantly impaired or abolished after ROFA or TiO_2_ exposure. A profound result of this study is that, despite our finding that ROFA or TiO_2_ exposure at 0.25 mg/rat did not cause significant pulmonary inflammation or damage (as judged by BAL PMNL influx, BAL albumin, and LDH release; Table 3), complete impairment of systemic A23187-induced endothelium-dependent arteriolar dilation was observed.

Second, pulmonary PM exposure appears to abolish both NO-dependent and NO-independent systemic arteriolar dilation (Figure 4). In the spinotrapezius muscle of the normal rat, NO synthase inhibition reduced the effect of intraluminal A23187 infusion by approximately 50% (open bars vs. black bars). The remaining responsiveness to A23187 may be due to a variety of NO-independent factors such as bradykinin, cyclooxygenase products, or ADO. Because the arteriolar response to intraluminal A23187 infusion after ROFA exposure is significantly less than that during L-NMMA superfusion in normal rats (blue bars vs. black bars), it appears that ROFA has the capacity to impair both NO-dependent and NO-independent arteriolar dilation.

Third, pulmonary PM exposure does not affect systemic arteriolar smooth muscle responsiveness to NO (Figure 5). Regardless of the iontophoretic ejection current, SNP applied to the arteriolar wall produced equivalent arteriolar dilation in both ROFA-exposed and saline-treated control rats. This finding is essential because it is impossible to characterize the adverse endothelial effects of PM exposure if the microvascular smooth muscle sensitivity to NO is not clearly defined.

Fourth, PM exposure increases the number of adhering and rolling leukocytes in spinotrapezius muscle venules (Figure 6). This significant increase in observable venular leukocytes (Figure 6C) could be due to *a*) an increased systemic leukocyte concentration and/or *b*) an increase in the adhesion activity between leukocytes and the venular endothelium. We did not observe a difference in the systemic leukocyte concentrations between ROFA-exposed and saline-treated control rats (Figure 6D). However, it is conceivable that the increase in venular leukocyte adhesion after ROFA exposure is sufficient to lower free circulating leukocytes, thereby preventing an accurate measurement of systemic leukocytes. This may account for the similar systemic leukocyte concentrations between saline-treated and ROFA-exposed rats.

Few studies have investigated the effect of acute PM exposure on vascular function. Inhalation of concentrated ambient particles (CAPs) and ozone in humans causes constriction of the brachial artery but has no effect on endothelium-dependent or -independent dilation (Brook et al. 2002). Our finding that endothelium-independent dilation is unaltered (Figure 5) after PM exposure is in agreement with Brook et al. (2002), but we did not observe an effect of PM exposure on resting arteriolar diameter (Table 2). However, because vascular tone reflects an integrated response to a variety of signals that vary in importance among vascular beds (Cornelissen et al. 2002; Hill et al. 2001; Zweifach 1991), our finding that arteriolar tone is unaltered after PM exposure is not necessarily at odds with the observations of Brook et al. (2002). Using flow-dependent dilation as an indirect index of endothelial function, Brook et al. (2002) found no effect of CAPs on endothelium-dependent dilation. In contrast, we delivered an agonist directly to the endothelium of a single arteriolar segment and found that endothelium-dependent dilation was potently impaired after PM exposure (Figures 2–4). A possible explanation for this discrepancy is that flow-dependent dilation activates endothelial NO synthase substantially through a Ca^2+^-independent pathway (Muller et al. 1999), but A23187 does so via a Ca^2+^-dependent pathway (Schneider et al. 2003). Additionally, the technique used to induce flow-dependent dilation (total flow stoppage by inflation of a cuff around the proximal forearm and subsequent release) would likely disturb the local environment, and the influence of local metabolites or myogenic activity under these conditions cannot be ruled out. The type of PM and exposure methods may also contribute to the discrepancies between our findings and those of Brook et al. (2002).

*In vitro* treatment of rat aortic rings and small arteries with PM stimulates vascular smooth muscle relaxation (Bagate et al. 2004; Knaapen et al. 2001). In these studies, the vessel segments were precontracted with phenylephrine and then exposed to a PM solution. PM exposure in this preparation induced a dose-dependent relaxation that was partially endothelium dependent. The tissue isolation required for *in vitro* experiments precludes any neural, local, physical, and/or hormonal influence on vascular physiology. *In vitro* observations on tissues exposed to PM may be of limited relevance because they mandate a direct interaction between PM and the systemic vasculature. In support of this possibility, PM has been reported to enter the systemic circulation. PM deposition in the arteriolar wall has been documented in stray Mexico City canines (Calderon-Garciduenas et al. 2001). Inhaled ultrafine ^13^C particles have been shown to translocate to extrapulmonary organs (Oberdorster et al. 2002). In contrast, studies with ultrafine ^192^Ir indicate that only a small fraction of inhaled ultrafine PM exits the lung (Kreyling et al. 2002).

Circulating leukocytes and platelets are known to increase after PM exposure (Salvi et al. 1999; Tan et al. 2000; Terashima et al. 1997). Up-regulation of bronchial adhesion molecules, such as ICAM-1 and VCAM-1, has also been documented after PM exposure (Salvi et al. 1999). However, the state of systemic microvascular adhesion molecules after PM exposure is unknown. Our observations that venular leukocyte adhesion and rolling are increased despite no change in the systemic leukocyte concentration after PM exposure (Figure 6) suggest that systemic adhesion molecules are up-regulated and/or activated. NO plays a vital role in protecting the endothelium from potential injury, as well as preventing inflammatory responses and atherosclerosis development (Cannon 1998). A loss of endothelial NO production could contribute to the altered venular leukocyte dynamics after PM exposure shown in Figure 6.

Tissue blood flow depends primarily on perfusion pressure and the caliber of resistance vessels. Changes in caliber are dictated by chemical and physical stimuli that originate within the immediate environment of resistance vessels and by changes in neural activity and circulating humoral agents (Shepherd 1983). Diminished tissue perfusion will disturb the local environment, most notably in the form of tissue hypoxia. Compensatory mechanisms, such as arteriolar dilation, attempt to restore normal tissue perfusion and therefore normoxia. Low arteriolar wall PO_2_ (partial pressure of O_2_) may serve as a stimulus for the release of endothelium-derived NO (Sauls and Boegehold 2000), which would dilate arterioles in an attempt to restore wall PO_2_. Typical responses such as these contribute to the maintenance of cardiovascular homeostasis under normal conditions. However, in diseased states such as hypercholesterolemia, hypertension, and coronary artery disease, endothelium-dependent dilation is often compromised (Drexler and Hornig 1999). If these populations respond to PM exposure as reported here, the impairment of endothelium-dependent dilation would compromise an already reduced vasodilator reserve, if not obliterate it, and may precipitate a cardiovascular incident.

In the present investigation, pulmonary exposure was accomplished by a single IT instillation of saline, ROFA, or TiO_2_. Some have questioned the relevance of such an exposure to the human condition of a continuous exposure by inhalation. A review sponsored by the Inhalation Specialty Section of the Society of Toxicology has addressed this issue (Henderson et al. 1995). Results indicate that pulmonary responses to particle exposure via inhalation versus IT instillation correlate well when lung burdens are equivalent. Additionally, our laboratory has shown that adverse response, that is, enhanced susceptibility to bacterial infection, is well distributed throughout the different regions of the lung after IT instillation of ROFA (Roberts et al. 2004). In light of such results, IT instillation has been judged as a useful exposure method for hazard identification and elucidation of mechanisms of toxic responses (Henderson et al. 1995).

Toxic pulmonary responses to ROFA have been linked to the high concentrations of soluble metals associated with these particles (Antonini et al. 2002; Dreher et al. 1997). In addition, ROFA particles are acidic, having a pH of approximately 4.1 suspended in saline. For these reasons, some have questioned whether ROFA particles are a good enough surrogate for ambient PM. Data from the present study indicate that pulmonary exposure to ROFA or TiO_2_ resulted in similar alterations of systemic microvascular function. These results argue against soluble metals being a prime factor in this response. Additionally, IT instillation of acidic saline solutions did not reproduce the pulmonary effects of ROFA (data not shown). Therefore, the effects of pulmonary exposure to ROFA on systemic microvascular function may be generalized to ambient PM.

In the present study, a significant impairment of systemic arteriolar dilation was observed 24 hr after IT instillation of 0.1 mg ROFA (Figure 2). How would this lung burden compare with that of humans exposed to ambient air? The current U.S. Environmental Protection Agency limit for ambient PM is 150 μg/m^3^ (U.S. EPA 1987). Levels in problem cities on high pollution days can easily exceed this limit by 3-fold. Assuming that *a*) the resting minute ventilation of 7.5 L/min means that 10.8 m^3^ of air is inhaled over a 24-hr period; *b*) 25% of inhaled PM with a mean diameter of approximately 2 μm would be deposited in the respiratory zone; and *c*) the alveolar surface area of humans is 250 times that of rats, pulmonary exposures in this study would exceed human exposures to ambient PM by 20-fold. Considering that special populations (i.e., the elderly and those with preexisting cardiovascular disease) exhibit increased susceptibility to PM, the results reported here using young healthy rats may be relevant.

## Conclusions

This study has identified significant effects of pulmonary PM exposure on the systemic microcirculation, which is the primary site of total peripheral resistance, tissue blood flow regulation, and plasma–tissue exchange. PM exposure studies in large conduit arteries are important, but the substantial anatomical and functional differences that exist between conduit arteries and arterioles preclude extrapolation of those findings to the microvascular level. Microvascular dysfunction is associated with essentially all cardiovascular diseases and is among the first targets of inflammatory and immune responses. Therefore, this study is a crucial and fundamental first step in identifying the mechanisms by which pulmonary PM exposure may increase morbidity and mortality. Results of the present study argue strongly for future investigations to *a*) identify the “no effect” lung burdens for ROFA and TiO_2_ for impairment of endothelium-dependent arteriolar dilation; *b*) determine what characteristics of ROFA are responsible for the impaired endothelium-dependent arteriolar dilation observed herein; *c*) determine the time course of microvascular effects after PM exposure; *d*) determine if other types of PM also elicit the same effect on the microvascular endothelium; and *e*) determine to what degree these observations are altered by age or pathologic states, such as hypertension, hypercholesterolemia, and diabetes, where microvascular structure and function are already compromised.

## Figures and Tables

**Table 1:** Profiles of experimental animals used for intravital studies (mean ± SE).

Experimental group	No. of rats	Age (days)	Weight (g)	Mean arterial pressure (mm Hg)
Saline	10	57 ± 4	233 ± 5	88 ± 6
0.25 mg TiO_2_	3	56 ± 3	219 ± 7	91 ± 15
0.1 mg ROFA	5	51 ± 1	223 ± 7	96 ± 9
0.25 mg ROFA	7	51 ± 3	209 ± 6	80 ± 6
1 mg ROFA	3	52 ± 3	238 ± 17	80 ± 6
2 mg ROFA	6	55 ± 4	218 ± 13	84 ± 5

**Table 2:** Resting variables for all arterioles studied (mean ± SE).

	Experimental group					
	Saline	0.25 mg TiO_2_	0.1 mg ROFA	0.25 mg ROFA	1 mg ROFA	2 mg ROFA
No. of arterioles	18	8	9	17	8	6
Resting diameter (μm)	43 ± 2	41 ± 2	41 ± 2	41 ± 1	44 ± 2	48 ± 4
Passive diameter (μm)	107 ± 4	100 ± 3	111 ± 6	100 ± 3	117 ± 5*	108 ± 5
Resting tone (percent of maximum)	59 ± 2	59 ± 2	62 ± 3	59 ± 1	62 ± 2	56 ± 3

* *p* < 0.05 vs. 0.25 mg TiO_2_ and 0.25 mg ROFA.

**Table 3:** BAL data in saline-, TiO_2_-, and ROFA-exposed rats (mean ± SE).

	Cellular content		BAL fluid		AM (CL)	
Experimental group	AM	PMNL	Albumin (mg/mL)	LDH (U/L)	Total CL	NO-dependent CL
Saline	6.20 ± 0.63	1.09 ± 0.12	0.12 ± 0.01	46 ± 5	8.87 ± 1.99	1.44 ± 0.35
0.25 mg TiO_2_	4.89 ± 0.65	1.11 ± 0.15	0.19 ± 0.02	65 ± 8	3.64 ± 0.73	0.42 ± 0.26
0.1 mg ROFA	5.22 ± 0.93	1.24 ± 0.28	0.20 ± 0.04	75 ± 2*	14.47 ± 2.60	4.40 ± 0.95
0.25 mg ROFA	6.25 ± 0.76	1.91 ± 0.20	0.17 ± 0.01	46 ± 4*^##^*	17.42 ± 1.11	5.38 ± 0.20
1 mg ROFA	6.12 ± 0.94	2.87 ± 0.38*^,^**, #,##	0.46 ± 0.08*^,^**, #,##	97 ± 9*^,^**, #	10.47 ± 1.86	3.45 ± 1.01
2 mg ROFA	6.47 ± 0.76	4.02 ± 0.40*^,^**, #,##, †	0.36 ± 0.04*^,^**, #,##	84 ± 9*, #	26.26 ± 7.14*^,^**, ##, †	9.81 ± 3.77*^,^**, #,##, †

CL, chemiluminescence. The number of rats in experimental groups were as follows: 17 for saline, 6 for TiO_2_, and 5 for all ROFA doses. We evaluated 10^6^ cells/rat for AM and for PMNL. Total CL and NO-dependent CL were determined as follows: counts per minute × 10^5^/0.25 × 10^6^ AM/15 min.

* *p* <0.05 vs. saline.

** *p* < 0.05 vs. TiO_2_.

# *p* < 0.05 vs. 0.25 mg ROFA.

## *p* < 0.05 vs. 0.1 mg ROFA.

† *p* < 0.05 vs. 1 mg ROFA.
